# The Second Intracellular Loop of the Human Cannabinoid CB2 Receptor Governs G Protein Coupling in Coordination with the Carboxyl Terminal Domain

**DOI:** 10.1371/journal.pone.0063262

**Published:** 2013-05-07

**Authors:** Congxia Zheng, Linjie Chen, Xiaopan Chen, Xiaobai He, Jingwen Yang, Ying Shi, Naiming Zhou

**Affiliations:** 1 Institute of Biochemistry, College of Life Sciences, Zhejiang University, Hangzhou, Zhejiang, China; 2 Institute of Sericulture and Apiculture, College of Animal Sciences, Zhejiang University, Hangzhou, Zhejiang, China; 3 School of Art, Zhejiang International Studies University, Hangzhou, Zhejiang, China; University of São Paulo, Brazil

## Abstract

The major effects of cannabinoids and endocannabinoids are mediated via two G protein-coupled receptors, CB1 and CB2, elucidation of the mechanism and structural determinants of the CB2 receptor coupling with G proteins will have a significant impact on drug discovery. In the present study, we systematically investigated the role of the intracellular loops in the interaction of the CB2 receptor with G proteins using chimeric receptors alongside the characterization of cAMP accumulation and ERK1/2 phosphorylation. We provided evidence that ICL2 was significantly involved in G protein coupling in coordination with the C-terminal end. Moreover, a single alanine substitution of the Pro-139 in the CB2 receptor that corresponds to Leu-222 in the CB1 receptor resulted in a moderate impairment in the inhibition of cAMP accumulation, whereas mutants P139F, P139M and P139L were able to couple to the G_s_ protein in a CRE-driven luciferase assay. With the ERK activation experiments, we further found that P139L has the ability to activate ERK through both G_i_- and G_s_-mediated pathways. Our findings defined an essential role of the second intracellular loop of the CB2 receptor in coordination with the C-terminal tail in G protein coupling and receptor activation.

## Introduction

Cannabis has been used in different civilizations for a variety of medical applications such as appetite stimulation and the treatment of pain, nausea, fever, and gynecological disorders for thousands of years [1], [2]. The cellular mechanism of action of cannabinoid drugs became clear with the discovery of cannabinoid binding sites in the brain and the subsequent cloning of the CB1 receptor [3], [4]. A second cannabinoid receptor, CB2, was identified from a human leukocyte cell line [5] and was once considered to be the “peripheral cannabinoid receptor” based on its abundant expression in the immune system, in contrast to the “central cannabinoid receptor” CB1, which is predominantly expressed in the central nervous system [6]. However, recent evidence has shed light on the role of the CB2 receptor in a variety of systems that now includes the CNS, as well as the peripheral immune system, the immune system of the CNS, the cardiovascular and respiratory systems, bone, the gastrointestinal (GI) tract, the liver and the reproductive system [7], [8], [9], [10]. Because the CB2 receptor is an attractive therapeutic target for pain management, immune-modulators and the treatment of liver diseases, an understanding of the mechanism and structural determinants of CB2 receptor coupling with G proteins will have a significant impact on drug discovery.

The cannabinoid receptors, CB1 and CB2, are members of the G protein-coupled receptor superfamily, and both the CB1 and CB2 receptors have been demonstrated to inhibit adenylyl cyclase activity through a pertussis toxin-sensitive G protein that leads to a decrease of cAMP levels in the cells. Unlike the CB1 receptor that has been shown to be capable of coupling to G_s_ in some cases [11], [12], [13], the CB2 receptor has not been found to couple with other G proteins [14]. CB2 receptor stimulation leads to activation of ERK1/2 MAP kinase via the Raf and PKC pathways in transfected CHO cells, HL60 cells, and prostate epithelial cells [15], [16], [17]. In neurons, the CB2 receptor activates the PI3K/Akt signaling pathway to protect cells from apoptosis upon stimulation [18]. In contrast to CB1, conflicting data on CB2-mediated modulation of calcium channels or inward rectification of potassium channels have been reported [19], [20]. Interestingly, in Jurkat T cells, JWH-015-mediated CB2 activation led to an initial decrease followed by a sustained and profound increase in cAMP production [21]. The increased cAMP resulted in suppression of T cell receptor signaling through a cAMP/PKA/Csk/Lck pathway [21]. However, the mechanism that caused the cAMP increase is still unknown.

In our previous study [22], we used different cell lines including HEK293, CHO, COS-7, 3T3 and HeLa cells that were expressing human CB1 or CB2 receptors to show that the CB1 receptor dually couples to the G_s_-mediated cAMP accumulation pathway and the G_i_-induced pertussis toxin (PTX)-sensitive activation of ERK1/2 and Ca^2+^ mobilization, whereas the CB2 receptor only couples to G_i_ and mediates an inhibitory effect on cAMP production. Using CB1/CB2 chimeric constructs and site-directed mutagenesis approaches combined with functional studies, we have identified an important role of the second intracellular loop and, in particular, residue Leu-222 as a critical mediator of G protein-coupling selectivity for the CB1 receptor [22]. In the present study, to gain insights into the detailed structural elements involved in the selective interaction of the CB2 receptor with either G_i_- or G_s_-proteins, we used the same approaches to characterize the intracellular loops and residues that contribute to the specific interaction of the CB2 receptor with G proteins. We demonstrated that the coordination of the second intracellular loop and the carboxyl terminal domain plays an essential role in the regulation of coupling of the human cannabinoid CB2 receptor with G proteins.

## Materials and Methods

### Materials

Cell culture media and G418 were purchased from Invitrogen (Carlsbad, CA). The pCMV-Flag vectors, forskolin (FSK) and pertussis toxin (PTX) were purchased from Sigma (St. Louis, MO). The pEGFP-N1 vector was purchased from Clontech (Mountain View, CA). Primary antibodies for Western blotting were purchased from Cell Signaling (Danvers, MA). WIN55212-2 (WIN) and H89 were obtained from Tocris (Ellisville, MO).

### Molecular Cloning, Plasmid Construction and Mutagenesis of Human CB1 and CB2

CB1 (GenBank Accession NM_016083.4) and CB2 (GenBank Accession NM_001841.2) receptors were cloned by PCR using human genomic DNA as a template. The PCR products were inserted into the *HindIII* and *BamHI* sites of the pCMV-Flag and pEGFP-N1 vectors. All constructs were sequenced to verify that they had the correct sequences and orientations. CB2/CB1 receptor chimeras were constructed by the exchange of restriction fragments between CB2 and CB1, using overlap extension PCR strategies. Point mutations were introduced into the CB2 receptor in the second intracellular loop by PCR overlap extension. Sequence analysis was performed to exclude frame shifts or point mutations and to control deletion of the termination codon. All of the constructs were generated by ligation of the chimeric receptors or mutated receptors into the *HindIII/BamHI* sites of the pCMV-Flag and pEGFP-N1 vectors.

### Cell Manipulation and Transfection

The HEK293 cell lines were maintained in Dulbecco’s Modified Eagles Medium (DMEM, Invitrogen) supplemented with 10% heat-inactivated fetal bovine serum (Hyclone). The CB2 plasmid constructs were transfected into cells using Lipofectamine 2000 (Invitrogen) according to the manufacturer’s instructions. Two days after transfection, selection for stable expression was initiated by the addition of G418 (800 µg/ml).

### Luciferase Assay

After seeding cells in a 96-well plate overnight, HEK293 cells stably or transiently cotransfected with Flag-CB2 and pCRE-Luc were grown to 90–95% confluence, stimulated with the indicated concentration of drug in DMEM without FBS, and incubated for 4 h at 37°C. Luciferase activity was detected by use of a firefly luciferase assay kit (Kenreal, Shanghai, China). When required, cells were treated overnight with PTX (100 ng/ml) or 1 hour with inhibitor in serum-free DMEM before the start of the experiment.

### ERK1/2 Activation

The HEK293 cells stably or transiently transfected with Flag-CB2 receptors were seeded in 12-well plates and starved for 4 h in serum-free medium to reduce background ERK1/2 activation. After stimulation with the drug, cells were lysed by the addition of lysis buffer [20 mM HEPES (pH 7.5), 10 mM EDTA, 150 mM NaCl, 1% Triton X-100, and one tablet of complete protease inhibitor (Roche, Indianapolis, IN) per 50 ml] at 4°C on a rocker for 30 min. The lysates were centrifuged at 4°C at 12,000 rpm for 15 min. Equal amounts of total cell lysate were size-fractionated by SDS-PAGE (10%) and transferred to a PVDF membrane (Millipore, Bedford, MA). Membranes were blocked in blocking buffer (TBS containing 0.05% Tween 20 and 5% non-fat dry milk) for 1 h at room temperature and then incubated with rabbit monoclonal anti-pERK1/2 antibody (Cell Signaling, Danvers, MA, USA) and anti-rabbit HRP-conjugated secondary antibody (CHEMICON, Temecula, CA, USA) according to the manufacturers’ protocols. Total ERK1/2 (Cell Signaling) was assessed as a loading control after p-ERK1/2 chemiluminescence detection using HRP substrate purchased from Cell Signaling. All the immunoblots were visualized and quantified by Bio-Rad Quantity One Imaging system (Bio-Rad Laboratories).

### ELISA Analysis of Cell-surface Expression

The CB2 receptors were analyzed for their comparative ability to traffic to the cell surface using enzyme-linked immunosorbent assay to detect the surface expression of the engineered Flag-tag epitope. HEK293 cells were seeded in poly-L-lysine treated 48-well plates and transfected using Lipofectamine 2000 as described above. The cells were fixed in 3.7% formaldehyde/TBS for 5 min at RT. The cells were then washed three times with TBS and nonspecific binding blocked with TBS containing 1% BSA for 45 min at RT. The first antibody (anti-Flag M2 monoclonal antibody, sigma) was added at a dilution of 1∶5000 in TBS/BSA for 1 h at RT. Three washes with TBS followed, and cells were briefly reblocked for 15 min at RT. Incubation with rabbit anti-mouse conjugated horseradish peroxidase (Sigma) diluted 1∶5000 in TBS/BSA was carried out for 1 h at RT. The cells were washed three times with TBS and a colorimetric peroxidase substrate was added. When the adequate color change was reached, 100 µl samples were taken for colorimetric readings. Cells transfected with pCMV-Flag were studied concurrently to determine background.

### Statistical Analysis

For ELISA analysis of cell-surface expression, the raw values of wild-type CB2 expression level were first averaged and normalized to 100% value, and then SEMs were calculated from the percent error on the 100% value. The CB2 mutants’ expression level was normalized to percentage of the wild type CB2 (wt). For cAMP experiments, all the raw data including control values were normalized and expressed as % values (%, referred to % of maximal, % of control, % of FSK and % of wild type receptor). Curves were fitted to a concentration-response curve to calculate the maximum response and −logEC_50_ (pEC_50_). Statistical analysis was performed by a one-way ANOVA, followed by Bonferroni post hoc test using Prism 5 Software (Graph-pad software, San Diego, CA). One-way ANOVA were performed using normalized values, p values of <0.05 were considered to indicate a significant difference.

## Results

### Agonist-induced Inhibition of Adenylyl Cyclase in Cells Expressing Human CB2 Receptors

**Figure 1:**
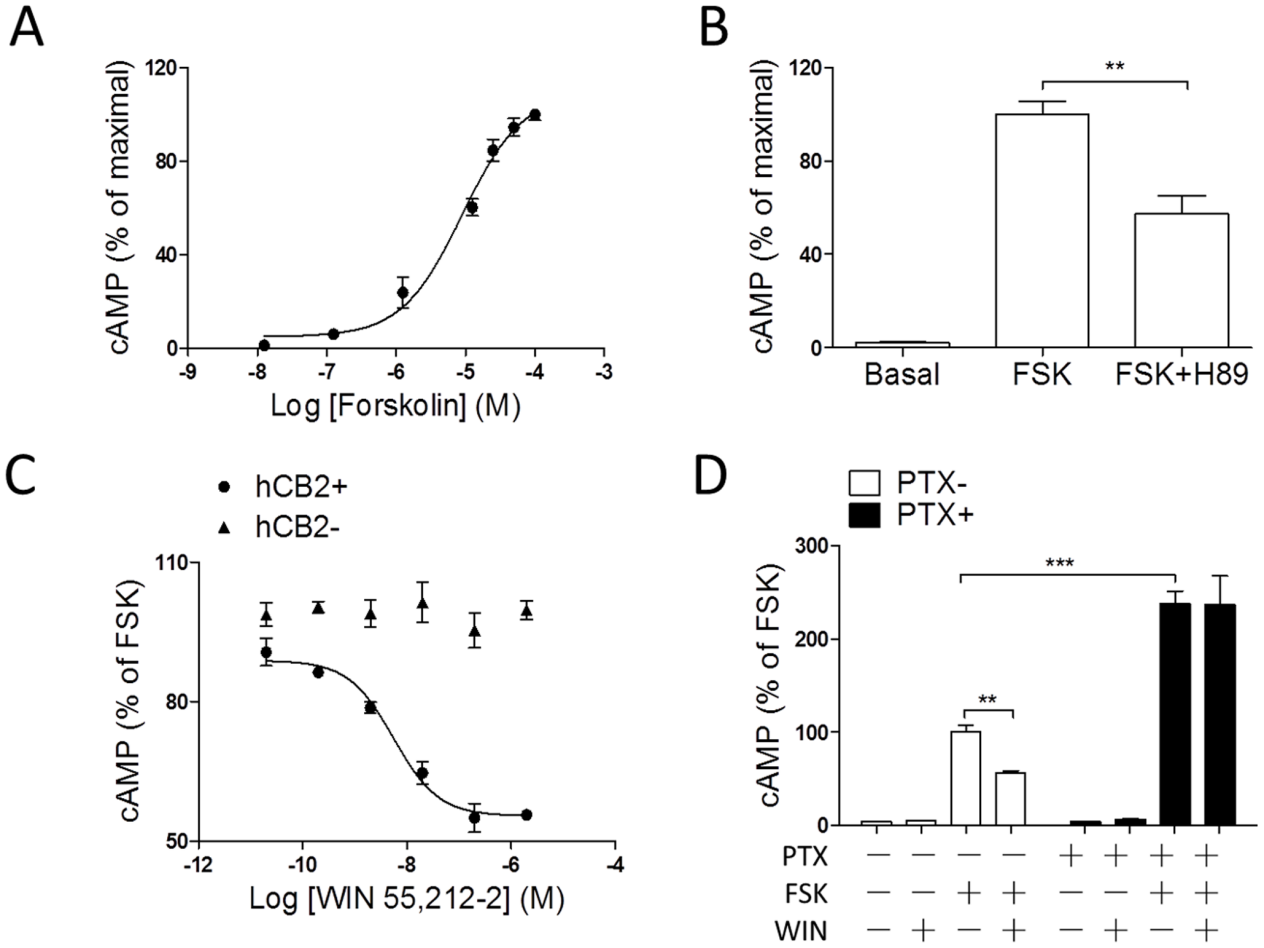
Agonist-induced inhibition of adenylyl cyclase in cells expressing the human CB2 receptor. (**A**) Characterization of cAMP signaling using CRE-luciferase assay. HEK293 cells transiently transfected with CRE-Luciferase were stimulated with various concentration of forskolin for 4 h. The CRE-driven luciferase activity obtained at 10^−4^ M forskolin stimulation was normalized to 100% value. (**B**) Effects of PKA inhibitor H89 (10 µM) on blockage of CRE-Luciferase activation induced by forskolin. HEK293 cells transiently expressing CRE-luciferase were pretreated with inhibitor for 1 h and stimulated with 10 µM forskolin for 4 h. The CRE-driven luciferase activity obtained at 10 µM forskolin stimulation was normalized to 100% value. (**C**) Dose-dependent curve of WIN55,212-2-mediated inhibition of forskolin-induced cAMP elevation. Cells transiently expressing CB2 receptor were incubated with 10 µM forskolin or 10 µM forskolin plus WIN55,212-2 (various concentrations) for 4 h. (**D**) Effects of PTX on cAMP accumulation of stable cell line HEK293-CB2 cells. Cells were seeded for 24 h prior to the addition of toxins. PTX (100 ng/ml) was added to the cells in FBS-free medium and cells were incubated for another 12 h. Cells were then incubated with 10 µM forskolin or 1 µM WIN55,212-2 plus 10 µM forskolin for 4 h. Data are expressed as the percent cAMP activity over forskolin. cAMP measurements were carried out as described in the Materials and Methods. Data are expressed as the mean ± SEM and are representative of three independent experiments. **p<0.01; ***p<0.001.

In our initial study, the stable HEK293 cell lines that express the human cannabinoid CB2 receptor and a reporter gene consisting of the firefly luciferase coding region under the control of a minimal promoter containing cAMP-response elements (CREs) were established for a quantitative analysis of intracellular cAMP changes. A dose-dependent luciferase activity was observed in response to forskolin with the maximal induction seen at approximately 100 µM and the half-maximal induction seen at approximately 10 µM (Fig. 1A), which is comparable to what was obtained from a previous radioactive cAMP assay [23]. We further examined whether the cAMP-mediated protein kinase A (PKA) signaling pathway was responsible for the luciferase activity. As demonstrated in Fig. 1B, pretreatment of cells with the PKA inhibitor H89 resulted in a significant reduction in the forskolin-induced luciferase expression. These results suggest that the luciferase activity correlates well with the cAMP/PKA gene transcription pathway, and the CRE-luciferase assay offers an alternative to the functional and biochemical assays for the CB2 receptor, which is consistent with our previous study [22].

We next assessed the inhibitory effects of an agonist on forskolin-induced intracellular cAMP accumulation. As shown in Fig. 1C, the non-selective cannabinoid agonist WIN55,212-2 exhibited an inhibitory effect on forskolin-stimulated cAMP accumulation in a dose-dependent manner with an pEC_50_ value of 8.23 in the CB2- expressing HEK293 cells, but not in non-transfected HEK293 cells. The WIN 55,212-2-induced inhibition of the forskolin-stimulated cAMP increase could be completely blocked by pretreatment with 100 ng/ml PTX for 12 h (Fig. 1D), suggesting the involvement of the G_i_ protein.

### Cell Surface Expression and Functional Characterization of CB1/CB2 Chimeric Receptors

**Figure 2:**
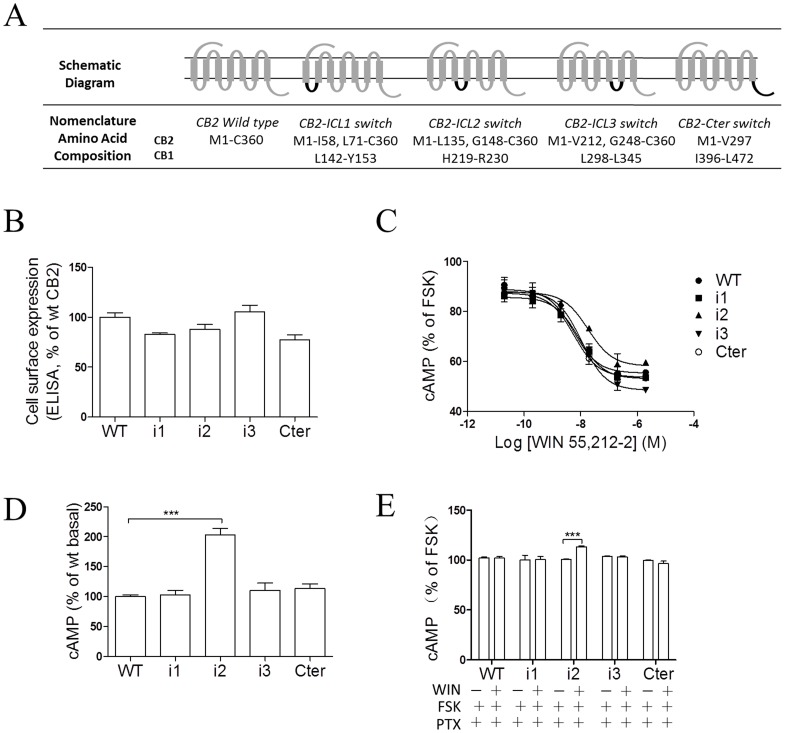
Effects of key domains in the CB2 receptor on G_i_-dependent signaling. (**A**) Schematic diagram of composition of cannabinoid CB2 receptor chimeras. The overall composition of individual cannabinoid receptor chimeras is shown schematically. Numbers indicate the amino acid residues corresponding to the parental cannabinoid receptors. The CB2 receptor sequence is shown in dark grey, and the CB1 receptor sequence is in black. (**B**) ELISA analysis of CB2 receptors expression. HEK293 cells were transiently transfected with Flag epitope-tagged receptors and the cell surface expression was measured by ELISA analysis, as described under *Methods*. The results represent the mean ± SEM of three independent experiments, each done in triplicate. (**C**) Dose-response curve of cAMP accumulation for the CB2 chimeric receptors upon agonist stimulation. For cAMP measurements, cells were incubated with various concentrations of WIN55,212-2 plus 10 µM forskolin for 4 h. cAMP measurements were carried out as described in the *Materials and Methods.* Data are expressed as the percent cAMP activity over forskolin. Data shown are expressed as the mean ± SEM and are representative of three independent experiments. (**D**) Basal cAMP accumulation of CB2 wild type and chimera receptors. For basal cAMP measurements, cells were incubated for 48 h after transfection and then directly lysed for cAMP assay. (E) Effects of PTX on cAMP accumulation of CB2 wild-type and chimera receptors. Cells were transiently transfected with receptors and pCRE-luc. 48 h later, PTX (100 ng/ml) was added to the reseeded cells in FBS-free medium and cells were incubated for another 12 h. Then, cells were incubated with 10 µM forskolin or 2 µM WIN55,212-2 plus 10 µM forskolin for 4 h and lysed for cAMP assay. Data shown are expressed as the mean ± SEM and are representative of three independent experiments. Data are expressed as the percent cAMP activity over wild type CB2 receptor. ***p<0.001. i1, CB2-ICL1 chimera receptor; i2, CB2-ICL2 chimera receptor; i3, CB2-ICL3 chimera receptor; Cter, CB2-Cter chimera receptor.

In a previous study [22], we demonstrated that the CB1 receptor is capable of dually coupling to the G_s_-mediated cAMP accumulation pathway and the G_i_-induced, PTX-sensitive activation of ERK1/2 and Ca^2+^ mobilization. As shown above, the CB2 receptor selectively couples to G_i_ and mediates an inhibitory effect on cAMP production. To determine the intracellular domains responsible for the selective activation of G_i_ by the CB2 receptor, chimeric cannabinoid receptors (CB2-ICL1, CB2-ICL2, CB2-ICL3, CB2-Cter) were constructed in which intracellular domains of the CB2 receptor were replaced with the corresponding segments of the CB1 receptor (Fig. 2A). These chimeric receptors were then expressed in HEK293 cells and characterized for cell surface expression and binding. As shown in Fig. 2B and Table 1, although the three chimeras, including CB2-ICL1, CB2-ICL2 and CB2-Cter, displayed a moderate decrease in membrane expression compared with the wild-type, the correct localization of CB2 and mutant receptors at the plasma membrane was further verified by visualization of EGFP-fused receptors with fluorescent microscopy (Fig. S1).

**Table 1:** Functional characterization of cannabinoid receptor chimeras and mutants.

	cAMP accumulation			
CB2 receptors	Basal (% of wt basal)	Inhibition rate (% of maximal)	Fold increase	pEC_50_ (EC50 (nM))
wt	100.0±3.1α	44.3±0.9	–	8.23±0.18 (7.0±2.9)
i1	102.4±8.1α	46.3±0.7	–	8.17±0.19 (8.0±2.8)
i2	203.1±10.5α	40.3±0.9	–	7.74±0.09 (19.2±4.4)
i3	110.3±12.4α	51.4±0.5	–	7.99±0.22 (13.4±7.3)
Cter	113.5±7.9α	46.5±0.8	–	8.23±0.03 (5.9±0.4)
i2i3	101.2±3.8α	44.7±1.2	–	8.47±0.15 (3.8±1.2)
i2i3Cter	97.8±8.9α	41.3±2.2	–	8.48±0.22 (4.4±2.4)
P139A	119.3±12.1α	41.2±0.8	–	7.9±0.21 (15.9±8.0)
i2Cter	172.1±13.4α	–	2.7±0.2α,b	6.85±0.1 (149±35)α,b
P139L	193.9±3.4α	–	4.2±0.3α,b	7.48±0.25 (45±2.3)α,b
P139F	207.6±8.2α	–	5.4±0.5α,b	8.28±0.19 (5.7±2.8)α,b
P139M	132.9±15.1α	–	4.8±0.5α,b	7.0±0.14 (103±31)α,b
P139LCter	209.6±5.3α	–	4.4±0.3α,b	7.53±0.11 (29.8±7.1)α,b
P139I	107.2±13.1α	–	1.0±0.1α,b	ND
P139V	111.2±4.1α	–	1.1±0.1α,b	ND

The values are expressed as the mean ± SEM (*n = *3 experiments). % of maximal, the value of cAMP level percentage of the value obtained upon 10 µM forskolin treated only. Fold increase, the valve of cAMP level related to basal activity.

ND, not detectable.

α The values were obtained in the absence of forskolin.

b The values were obtained in the presence of 2 µM WIN55,212-2.

HEK293 cells expressing four CB2 chimera receptors and wild-type CB2 receptor were functionally determined by the CRE-luciferase assay. The chimeric mutants with a substitution of the corresponding segments of the CB1 receptor resulted in an effective inhibition of forskolin-mediated stimulation of adenylyl cyclase activity that was comparable to the wild-type receptor, while only the chimera CB2-ICL2 showed a decreased inhibition in luciferase activity with a significantly reduction in pEC_50_ (Fig. 2C and Table 1). Our results showed that the CB2 chimera containing the second intracellular loop of CB1 exhibited a two-fold increase in basal activity as compared to the wild-type and other CB2 chimeras (Fig. 2D). Then, we examined the effect of PTX pretreatment on the agonist-stimulated cAMP accumulation of the CB2 chimeras. As shown in Fig. 2E, the chimeric CB2-ICL2 receptor exhibited a significant CRE-driven luciferase activity, whereas wild-type and other chimeras showed no potential in agonist-mediated cAMP formation. Taken together, our data demonstrated the possible role of the second intracellular loop in the CB2-G protein coupling.

### C-terminal Domain of CB1 Receptor is Required for CB2/CB1 Chimeric Receptors to Full Couple the G_s_ Protein

**Figure 3:**
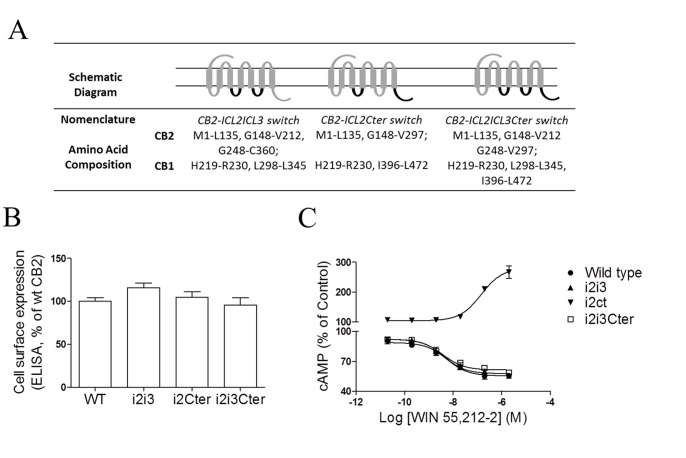
Effects of multi-domains in the CB2 receptor on G_s_- and G_i_-dependent signaling. (**A**) Schematic diagram of composition of CB2 chimeras with multi-domains substitution. The overall composition of individual cannabinoid receptor chimeras is shown schematically. Numbers indicate the amino acid residues corresponding to the parental cannabinoid receptors. The CB1 receptor sequence is shown in black, and the CB2 receptor sequence is in dark grey. (**B**) ELISA analysis of CB2 receptors expression. HEK293 cells were transiently transfected with Flag epitope-tagged receptors and the cell surface expression was measured by ELISA analysis, as described under *Methods*. The results represent the mean ± SEM of three independent experiments, each done in triplicate. (**C**) Effects of cAMP accumulation for multi-chimeric receptors upon agonist stimulation. For cAMP measurements, cells were incubated with 10 µM forskolin or with various concentrations of WIN55,212-2 plus 10 µM forskolin, except the chimera CB2-ICL2Cter in the absence of forskolin. For CB2-ICL2ICL3, CB2-ICL2ICL3Cter and CB2 wild-type, values are expressed as percentage of forskolin stimulation. For CB2-ICL2Cter, values are expressed as percentage of basal activity. i2i3, CB2-ICL2ICL3 chimera receptor; i2i3cter, CB2-ICL2ICL3Cter chimera receptor; i2Cter, CB2-ICL2Cter chimera receptor.

To further define the structural domains of the intracellular loops and the C-terminal that are required for CB2 to interact with the G protein, we constructed a series of CB2 chimeric mutants with a replacement of multiple intracellular domains, as illustrated in Fig. 3A. All of the tested multi-chimeras did not show any significant difference in the level of surface expression compared with the wild-type (Fig. 3B). The correct localization at the plasma membrane of these chimeras was further verified by visualization of EGFP-fused receptors with fluorescent microscopy (Fig. S2). Each chimeric mutant was then coexpressed with pCRE-Luc in HEK293 cells and assayed for WIN55,212-2-induced intracellular cAMP changes. The chimeric mutants CB2-ICL2ICL3 and CB2-ICL2ICL3Cter did not affect the ability of the CB2 receptor to interact with the G_i_ protein, characterized by inhibiting forskolin-stimulated cAMP accumulation with a similar maximum inhibition (44.7% and 41.3%, respectively) and a similar pEC_50_ value (8.47 and 8.48, respectively) in response to the stimulation of agonist WIN55,212-2 (Fig. 3C and Table 1). In contrast, as illustrated in Figure 3C and Table 1, the double-chimeric CB2 mutant (CB2-ICL2Cter) in which the second intracellular loop and C-terminal was replaced with the corresponding CB1 receptor sequence, gained the ability to stimulate cAMP production with a maximal stimulation (2.7 fold) and a significantly low pEC_50_ value (6.85) upon exposure to agonist WIN 55,212-2. These results suggest a role of the second intracellular loop and C-terminal tail in coordinating CB2 receptor interaction with G proteins.

### Proline-139 is a Key Residue Involved in the Interaction of the CB2 Receptor with G Proteins

**Figure 4:**
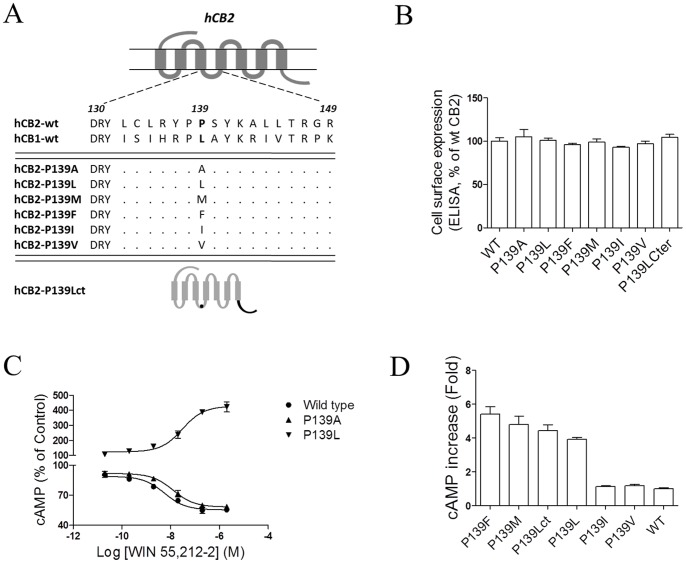
Effects of key residues in the ICL2 of the CB2 receptor on selectively G_i_ and G_s_ coupling. (**A**) Structures of CB2 mutations within the second intracellular loop as well as the C-terminal. (**B**) ELISA analysis of CB2 receptors expression. HEK293 cells were transiently transfected with Flag epitope-tagged receptors and the cell surface expression was measured by ELISA analysis, as described under *Methods*. The results represent the mean ± SEM of three independent experiments, each done in triplicate. (**C**) Dose response curve of cAMP accumulation for the CB2P139A and CB2P139L upon WIN55,212-2 stimulation. For cAMP measurements, cells were incubated with various concentrations of WIN55,212-2 for P139L and with various concentrations of WIN55,212-2 plus 10 µM forskolin for P139A and wild-type for 4 h. Values were expressed as percentage of forskolin stimulation for CB2P139A and CB2 wild-type, and as percentage of basal activity for CB2P139L. (**D**) Effects of substitutions of P139 with various kinds of amino acids in the CB2 receptor on WIN55,212-2-induced cAMP formation. HEK293 cells were treated with 2 µM WIN55,212-2, and cAMP production was measured as described in the *materials and methods*. The resulting increases in cAMP were expressed as fold increase above basal. Data are expressed as the mean ± SEM and are representative of three independent experiments.

Our previous study demonstrated that the residue Leu-222 of the CB1 receptor, which resides within a highly conserved DRY(X)_5_PL motif, plays a critical role in the receptor coupling with the G_s_ and G_i_ proteins [22]. To further define the role of the CB2 receptor residue Pro-139, corresponding to the CB1 receptor residue Leu-222, in G protein coupling, we next mutated Leu-222 to a number of different residues including Ala, Leu, Met, Phe, Ile, and Val (Fig. 4A). We determined that the mutants retained a similar cell surface expression relative to wild-type (Fig. 4B and Fig. S3). Each receptor mutant was coexpressed with pCRE-Luc in HEK293 cells for further analysis by a functional assay. As shown in Figure 4C, the potency and efficacy of the alanine substitution mutants P139A on the inhibition of forskolin-induced cAMP formation in response to WIN55,212-2 were slightly impaired compared with wild-type CB2; whereas the mutants with the replacement of Pro with Leu, Met, and Phe exhibited a stimulatory effect on intracellular cAMP production in response to agonist treatment with an enhancement of 4.0-, 4.8-, and 5.4-fold, respectively, in CRE-driven luciferase activity (Fig. 4D).

To further evaluate the role of the C-terminal tail in the interaction of the CB2 receptor with the G protein, we prepared a mutant construct of CB2 bearing the P139L site-mutation and the C-terminal tail of CB1. As shown in Fig. 4E and Table 1, the mutant expressed in HEK293 cells exhibited a stimulation of intracellular cAMP production (4.4 fold) comparable to the CB2 P139L (4.0 fold), indicating that the coordination of the amino acid Pro-139 located at the center of the ICL2 with the C-terminal tail promotes a more efficient interaction with the G protein.

### Distinct Activation of ERK1/2 Pathway by the Wild-type and the P139L Mutant CB2 Receptor

**Figure 5:**
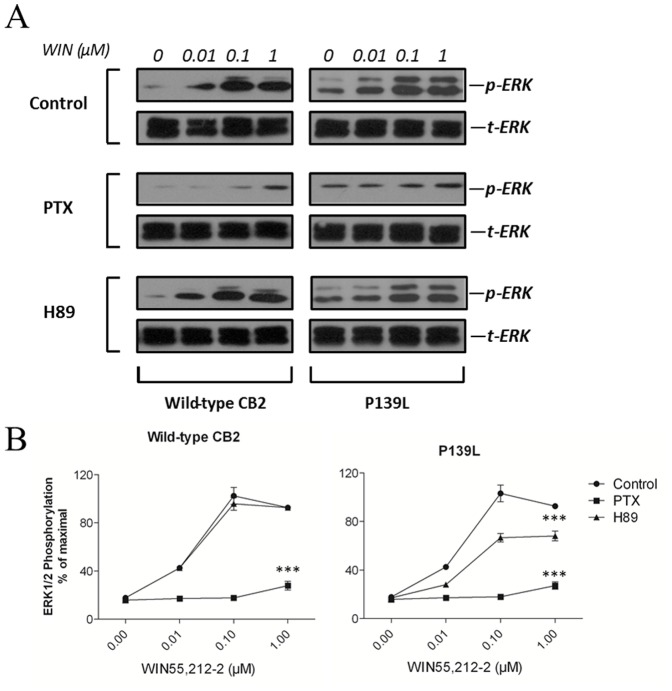
Comparison of effects of G_i_ inhibitor and PKA inhibitor on the activation of ERK in wild-type CB2 and P139L expressing cells. (**A**) Transiently transfected HEK293 cells were pretreated with or without 100 ng/mL *Pertussis toxin* (PTX) for 12 h or pretreated with or without 10 µM H89 for 30 min, and then stimulated with increasing concentrations of WIN55,212-2. (**B**) ERK signals were quantified by densitometry and expressed as a ratio of activated over total ERK. The maximal phosphorylation of ERK obtained in control cells at 5 min of stimulation with WIN55,212-2 in the absence of inhibitors were arbitrarily chosen as 100%. Each data point represents the mean ± SEM from three independent experiments. ***P<0.001 compared with the entire control curve.

It has been well established that the MAP kinase pathway has emerged as an important effector for G protein coupled receptors (GPCRs) and can be used to assess the functional outcome of receptor stimulation [24]. Therefore, we assessed the CB2-mediated activation of ERK1/2 in HEK293 cells stably expressing wild-type and mutant proteins using a phospho-specific antibody detecting the phosphorylated (Thr-202 and Tyr-204 of ERK1 and Thr-185 and Tyr-187 of ERK2) and activated forms of these kinases [25]. HEK293 cells that were transfected with wild-type CB2 or the P139L mutant were serum-starved overnight and then stimulated with increasing concentrations of WIN 55,212-2 for 5 min. As illustrated in Figure 5A and 5B, stimulation with agonist WIN55,212-2 elicited a transient phosphorylation of ERK1/2 but without a difference between wild-type CB2 and the P139L mutant. To explore the role of the G_i_-dependent pathway or G_s_/PKA pathway in the activation of ERK1/2, cells were pretreated with the G_i_ inhibitor PTX (100 ng/mL) overnight or 10 µM of the PKA inhibitor H89 for 30 min prior to stimulation with different concentrations of WIN55,212-2 and then were lysed for western blot analysis. As indicated in Figure 5A and 5B, pretreatment with PTX but not PKA inhibitor H89 was able to significantly abolish the activation of ERK1/2 in cells expressing wild type CB2. However, in cells expressing P139L mutant CB2, PTX treatment led to a significant inhibition of WIN55, 212-2-mediated ERK1/2 activation, but pretreatment with H89 also resulted in a partial suppression of ERK1/2 phosphorylation. Taken together, these results demonstrate that wild-type CB2 exclusively activated the ERK1/2 pathway via G_i_-dependent pathways while the P139L mutant has the ability to activate ERK1/2 through both G_i_- and G_s_-mediated pathways.

## Discussion

The effects of cannabinoids are mediated by two types of cannabinoid receptors, CB1 and CB2. Both CB1 and CB2 receptors primarily signal through a pertussis toxin-sensitive G protein that leads to the inhibition of adenylyl cyclase [6]. The potentiation of cAMP production was also observed in response to cannabinoid agonists under conditions of PTX pretreatment in cultured neurons and CB1-transfected CHO cells and upon coexpression of CB1 with the D2 dopamine receptor in striatal cells and in HEK293 cells [11], [12], [13], which suggests that the CB1 receptor can also interact with G_s_ proteins. In our previous studies, we used receptor chimeras and site-directed mutagenesis strategies to demonstrate the evidence of interaction of the CB1 receptor with G_s_ proteins. Our additional functional assays revealed both G_s_ and G_i_ proteins are involved in the CB1-mediated signaling [22]. In this study, using a number of chimeric receptors produced by substitution of intracellular domains with the CB1-corresponding part and mutants with site-directed mutations within the ICL2, we showed that the chimeric receptor CB2-ICL2Cter exhibited a stimulation of the intracellular cAMP accumulation, suggesting that the coordination of the second intracellular loop with the C-terminal tail determines the interaction of the CB2 receptor with the G protein. Moreover, a CB2 mutant with a substitution of Pro-139 for Leu exhibited a stimulatory effect on cAMP production and both cAMP/PKA pathway- and G_i_-dependent pathway-mediated activation of ERK1/2. These results indicate that the residue Pro-139 within the highly conserved DRY(X)_6_P motif may be a key player in the interaction of the CB2 receptor with the G protein.

Previous studies have been performed to define the structural determinants of GPCRs in the interactions with G proteins using chimeric constructs and site-directed mutagenesis of receptors. These studies have provided increasing evidence to show that multiple intracellular regions including the second intracellular loop (ICL2), the membrane-proximal portions of the third intracellular loop (ICL3) and the N-terminal segment of the cytoplasmic tail are likely to define a domain on the intracellular surface of the receptor protein. This domain, following activation of the receptor by an agonist ligand, can productively interact with distinct G protein heterotrimers [26], [27]. However, numerous studies have established the pivotal role of the second intracellular loop (ICL2) in determining receptor and G protein coupling and interaction [28], [29], [30]. Replacement of the entire ICL2 of the bradykinin B2 receptor with the E2 prostaglandin receptor resulted in a cAMP-generating receptor, which indicated the importance of this domain for G_s_ coupling and activation [31]. For the AT1 angiotensin receptor [32], this domain appears to have a direct role in agonist-induced G protein coupling. In addition, the crystal structures of GPCRs provide us with an abundance of information on the relationship between the structure and function of a GPCR. Recent crystallographic studies have suggested that G protein activation requires a conformation change in the ICL2 [33], [34], [35]. In the present study, the substitution of the second intracellular loop of CB2 with the corresponding region of CB1 receptor displayed a two-fold increase in basal activity as compared to the wild-type and other CB2 chimeras, suggesting the possible role of ICL2 in the interaction of CB2 with G proteins. The chimeric mutant with a replacement of both the ICL2 and C-terminal tail with the corresponding regions of CB1 led to a switch of G protein coupling from G_i_ to G_s_. These results indicate that the ICL2 of CB2 is likely to play a key role in the specificity of G protein coupling in coordination with the C-terminal tail.

Moreover, the importance of several structural determinants present in the ICL2 on the interaction between the receptor and the G protein has been documented for a number of GPCRs [29], [36], [37]. In particular, the DRY(X)_6_L motif present in the majority of rhodopsin-like receptors has been implicated in the G protein interaction and receptor activation [38], [39]. An alignment of peptide sequences corresponding to the ICL2 region in other G protein-coupled receptors shows that most GPCRs of the rhodopsin family contain a relatively bulky lipophilic amino acid, such as isoleucine, phenylalanine, methionine, or valine, at the position of the last residue in the DRY(X)_6_L motif. Substitutions with alanine or hydrophilic amino acids in this residue in the M1 and M3 muscarinic receptors [39], the β2-adrenergic receptor [39], the gonadotropic-releasing hormone receptor [36], the serotonin_2C_ receptor [40], and mouse prostaglandin receptors EP2 and EP3 [41] revealed that the resulting mutant receptors were greatly impaired or enhanced in their ability to activate G proteins. A single leucine to serine (L148S) mutation in the last residue of the DRY(X)_6_L motif of human GPR45 causes idiopathic hypogonadotropic hypogonadism (IHH), a disorder characterized by delayed puberty and infertility. Further characterization of L148S hGPR54 revealed that conserved residues in the IL2 of the Class A GPCRs are essential for functional interactions between the GPCR and G proteins [42]. The residue Pro-139 of CB2 corresponds to the last residue of the DRY(X)_6_P motif. We demonstrated that the P139A CB2 mutant exhibited only a slightly decreased functional response to agonist WIN55,212-2, whereas the replacement of proline with the very hydrophobic amino acids leucine (P139L), methionine (P139M), and phenylalanine (P139F) caused a stimulation of intracellular cAMP accumulation to a different extent. These results suggest that Pro-139 in the highly conserved motif DRY(X)_6_P plays a critical role in the CB2 and G protein interaction and receptor activation, which is highly consistent with our previous observation for the CB1 receptor [22]. The first crystal structure of the β2AR-Gs complex revealed that the active β2AR–GasRas interface is formed by ICL2, TM5 and TM6 of the β2AR, and by α5-helix, the αN–β1 junction, the top of the β3-strand, and the α4-helix of GasRas, and the interaction of the β2AR with the GasRas involves F139, the residue corresponding to P139 of CB2 [43]. The β2AR mutant F130A has severely impaired coupling to Gs protein [39] **.** Taken together, the highly conserved motif DRY(X)_6_P is more likely to define the specificity of GPCR-G protein coupling.

Mitogen-activated protein kinase (MAPK) pathways regulate diverse processes ranging from proliferation and differentiation to apoptosis. It is now known that GPCRs regulate MAPK cascades via distinct G proteins, β-arrestin-dependent and EGFR transactivation signaling pathways that lead to activation of the extracellular signal-regulated kinases (ERKs), which function as transcriptional regulators [24]. Therefore, characterization of the signaling pathways that stimulate MAPK activation through a particular receptor is essential to understand its role in physiology and pathology. The CB2 receptor has been shown to activate p42/p44 MAP kinase in transfected CHO cells and HL60 cells, and the activation could be blocked with PTX and the CB2 antagonist SR144528 [15], [16]. In the present study, activated wild-type CB2 receptors triggered phosphorylation of ERK1/2 in HEK293 cells via a PTX-sensitive G_i_ protein pathway, whereas the P139L mutant caused the activation of the ERK1/2 pathway through both a predominant PTX-sensitive G_i_ protein pathway and to a lesser extent a PKA-dependent pathway in response to the agonist WIN 55,212-2, suggesting that the P139L mutant might dually couple to G_i_ and G_s_ proteins. Although we only observed the agonist-stimulated cAMP increase in cells expressing the P139L mutant using a CRE-driven reporter assay, it was likely that the G_i_-mediated inhibitory effect was masked by productive cAMP accumulation. This is in high agreement with our previous study on CB1 receptor [22]. Ramanjaneya et al. demonstrated that Gq-, Gs- and Gi-coupled orexin receptor-1 (OX1R) exhibited a predominantly Gq- and to a lesser extent a Gs-mediated ERK1/2 phosphorylation [44]. Previous study also showed that Gs-coupled glucagon-like peptide-2 receptor (GLP-2R) signals to ERK1/2 pathway via Gi/Go-coupled pathway in PTS-sensitive manner upon teatment of GLP-2 agonist [45].

In summary, our results have defined an essential role of the second intracellular loop of the CB2 receptor in coordination with the C-terminal tail in G protein coupling and receptor activation. Moreover, we have identified the residue Pro-139 in the highly conserved motif DRY(X)_6_P as a critical residue in the interaction of the CB2 receptor with G proteins. Our characterization of the CB2 receptor in the interaction with the G protein has revealed fundamental concepts concerning GPCRs and G protein coupling and signal transduction.

## Supporting Information

Figure S1 **Fluorescence microscopy analysis of CB2 chimera CB2ICL1, CB2ICL2, CB2ICL3 and CB2Cter expression and localization.** HEK293 cells were transiently transfected with EGFP-fused CB2 receptors and the cell surface expression was analyzed by fluorescence microscopy as described under Methods. The cells shown are representative of the cell populations and performed at least three times. WT, CB2 wild-type receptors; i1, CB2ICL1 chimera; i2, CB2ICL2 chimera; i3, CB2ICL3 chimera; Cter, CB2Cter chimera
(TIF)

Figure S2 **Fluorescence microscopy analysis of CB2 chimera CB2ICL2ICL3, CB2ICL2Cter and CB2ICL2ICL3Cter expression and localization.** HEK293 cells were transiently transfected with EGFP-fused receptors and the cell surface expression was analyzed by fluorescence microscopy as described under Methods. The cells shown are representative of the cell populations and performed at least three times. WT, CB2 wild-type receptors; i2i3, CB2ICL2ICL3 chimera; CB2 CB2ICL2Cter chimera; i2i3Cter, CB2ICL2ICL3Cter chimera.
(TIF)

Figure S3 **Fluorescence microscopy analysis of CB2 mutants CB2P139A, CB2P139L, CB2P139M, CB2P139F, CB2P139I, CB2P139V and CB2P139LCter expression and localization.** HEK293 cells were transiently transfected with EGFP-fused receptors and the cell surface expression was analyzed by fluorescence microscopy as described under Methods. The cells shown are representative of the cell populations and performed at least three times. WT, CB2 wild-type receptors; P139A, CB2 mutant CB2P139A; P139L, CB2 mutant CB2P139L; P139M, CB2 mutant CB2P139M; P139F, CB2 mutant CB2P139F; P139I, CB2 mutant CB2P139I; P139V, CB2 mutant CB2P139V; P139LCter, CB2 mutant CB2P139LCter.
(TIF)

Methods S1 **Fluorescence microscopy analysis.**
(DOC)
