# Correction: The Second Intracellular Loop of the Human Cannabinoid CB2 Receptor Governs G Protein Coupling in Coordination with the Carboxyl Terminal Domain

**DOI:** 10.1371/annotation/11146424-f236-4fc6-85b2-cd393cfb6a94

**Published:** 2013-11-07

**Authors:** Congxia Zheng, Linjie Chen, Xiaopan Chen, Xiaobai He, Jingwen Yang, Ying Shi, Naiming Zhou

There is an error in Reference 22. The correct reference is:

Chen XP, Yang W, Fan Y, Luo JS, Hong K, et al. (2010) Structural determinants in the second intracellular loop of the human cannabinoid CB(1) receptor mediate selective coupling to G(s) and G(i). Br J Pharmacol 161:1817 -1834 

