# Supplementary figures and images for: The Second Intracellular Loop of the Human Cannabinoid CB2 Receptor Governs G Protein Coupling in Coordination with the Carboxyl Terminal Domain
Source: PLoS One. 2013 May 7;8(5):e63262. doi: 10.1371/journal.pone.0063262 (PMC3646771; doi:10.1371/journal.pone.0063262)

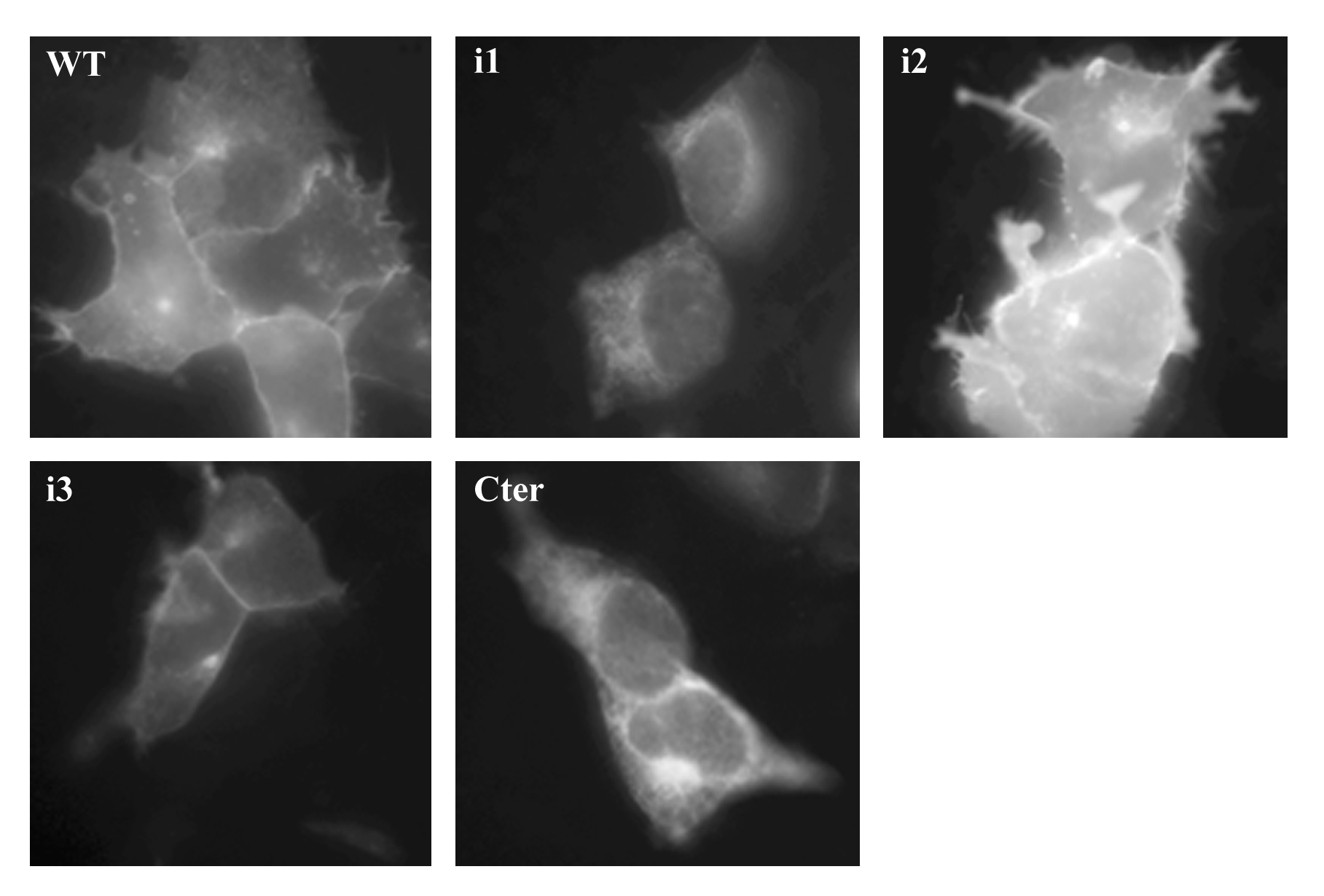

Supplement: Figure S1 — Fluorescence microscopy analysis of CB2 chimera CB2ICL1, CB2ICL2, CB2ICL3 and CB2Cter expression and localization. HEK293 cells were transiently transfected with EGFP-fused CB2 receptors and the cell surface expression was analyzed by fluorescence microscopy as described under Methods. The cells shown are representative of the cell populations and performed at least three times. WT, CB2 wild-type receptors; i1, CB2ICL1 chimera; i2, CB2ICL2 chimera; i3, CB2ICL3 chimera; Cter, CB2Cter chimera (TIF) [file pone.0063262.s001.tif]

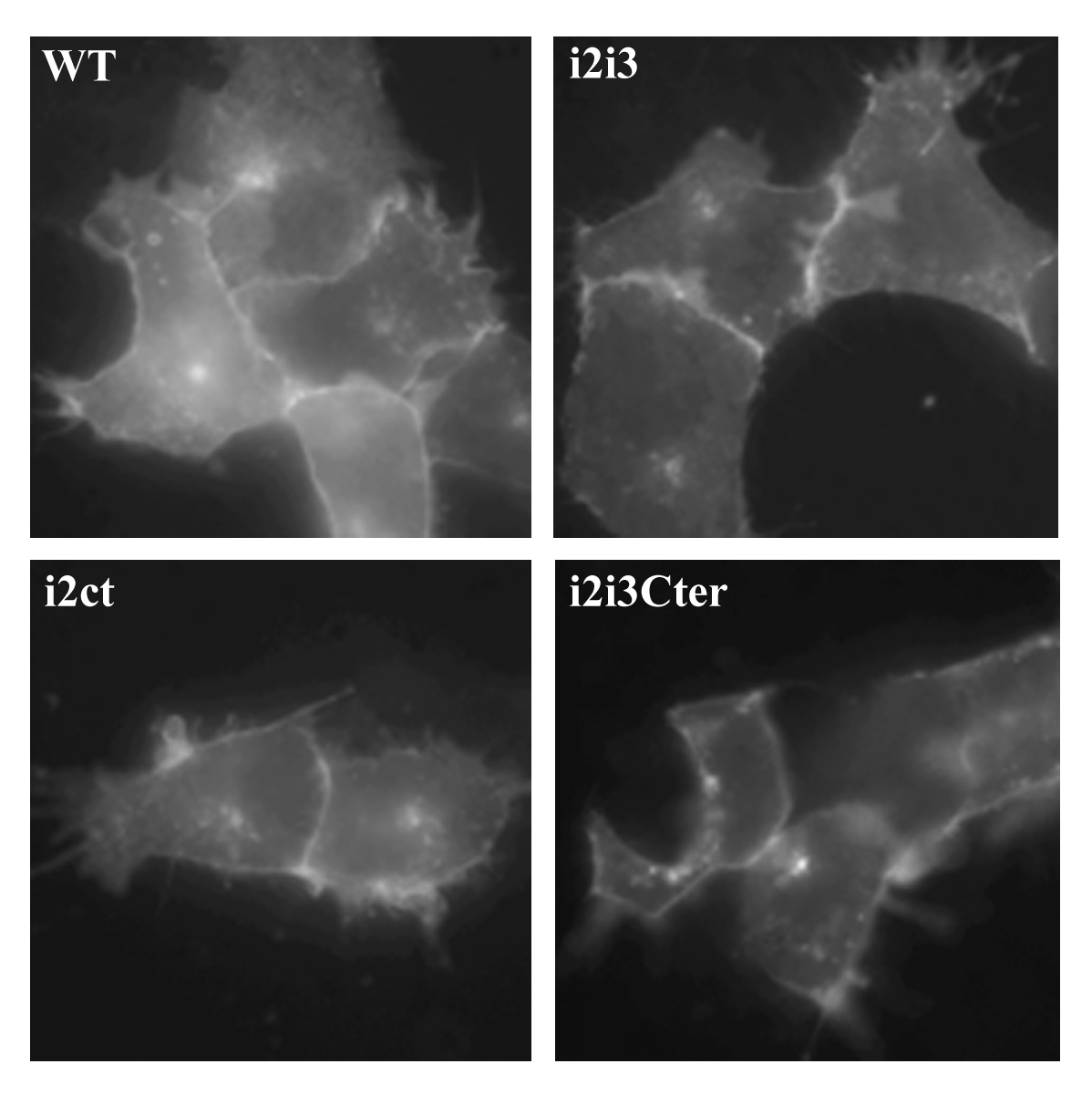

Supplement: Figure S2 — Fluorescence microscopy analysis of CB2 chimera CB2ICL2ICL3, CB2ICL2Cter and CB2ICL2ICL3Cter expression and localization. HEK293 cells were transiently transfected with EGFP-fused receptors and the cell surface expression was analyzed by fluorescence microscopy as described under Methods. The cells shown are representative of the cell populations and performed at least three times. WT, CB2 wild-type receptors; i2i3, CB2ICL2ICL3 chimera; CB2 CB2ICL2Cter chimera; i2i3Cter, CB2ICL2ICL3Cter chimera. (TIF) [file pone.0063262.s002.tif]

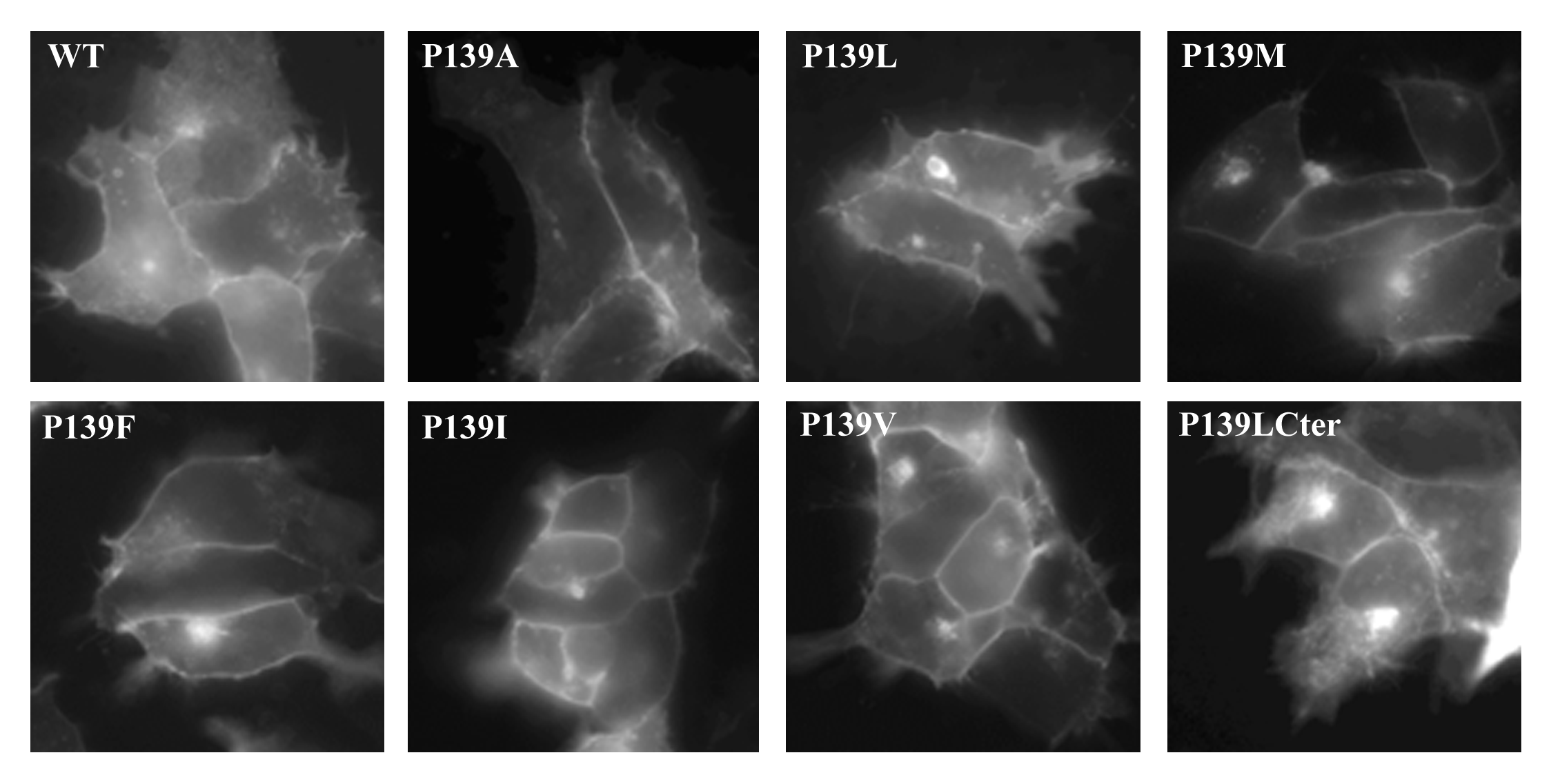

Supplement: Figure S3 — Fluorescence microscopy analysis of CB2 mutants CB2P139A, CB2P139L, CB2P139M, CB2P139F, CB2P139I, CB2P139V and CB2P139LCter expression and localization. HEK293 cells were transiently transfected with EGFP-fused receptors and the cell surface expression was analyzed by fluorescence microscopy as described under Methods. The cells shown are representative of the cell populations and performed at least three times. WT, CB2 wild-type receptors; P139A, CB2 mutant CB2P139A; P139L, CB2 mutant CB2P139L; P139M, CB2 mutant CB2P139M; P139F, CB2 mutant CB2P139F; P139I, CB2 mutant CB2P139I; P139V, CB2 mutant CB2P139V; P139LCter, CB2 mutant CB2P139LCter. (TIF) [file pone.0063262.s003.tif]
