# Supplementary material for: The Second Intracellular Loop of the Human Cannabinoid CB2 Receptor Governs G Protein Coupling in Coordination with the Carboxyl Terminal Domain
Source: PLoS One. 2013 May 7;8(5):e63262. doi: 10.1371/journal.pone.0063262 (PMC3646771; doi:10.1371/journal.pone.0063262)
Supplement: Methods S1 — Fluorescence microscopy analysis. (DOC) [file pone.0063262.s004.doc]

**Methods S1. Fluorescence microscopy analysis.**

HEK293 cells transiently transfected with CB2-EGFP and mutant-EGFP were seeded in cover glass-bottomed six-well plates. Forty-eight hours after transfection, cells were washed with PBS and then fixed with 2% paraformaldehyde for 15 min at room temperature. Finally, cells were mounted in mounting reagent (dithiothreitol/PBS/glycerol). Images were acquired on an Olympus BX60 fluorescence microscope using an objective oil immersion lens, UPlanFl 100X. Image handling was assembled in Adobe Photoshop.
